# Association of plasma PCB levels and HbA1c concentration in Iran

**DOI:** 10.1186/s12995-018-0199-4

**Published:** 2018-05-31

**Authors:** Sahar Eftekhari, Omid Aminian, Zeinab Moinfar, Thomas Schettgen, Andrea Kaifie, Michael Felten, Thomas Kraus, André Esser

**Affiliations:** 1 Institute for Occupational, Social and Environmental Medicine, University Hospital Aachen, RWTH University Aachen, Pauwelsstr 30, D-52074 Aachen, Germany; 2 School of Medicine- International Campus, Tehran University of Medical Sciences (TUMS), Tehran, Iran; 3 Center for Research on Occupational Diseases, Tehran University of Medical Sciences (TUMS), Tehran, Iran; 4 Community and Preventive Medicine Department, Tehran University of Medical Sciences (TUMS), Tehran, Iran

**Keywords:** PCB, HbA1c, Glucose disturbance polychlorinated biphenyls; diabetes mellitus

## Abstract

**Background:**

The rapid increase in prevalence of diabetes mellitus over the last decades warrants more attention to the effects of environmental and occupational exposures on glucose metabolism. Our study aimed to assess the association between the plasma levels of various congeners of polychlorinated biphenyls (PCBs) and the serum concentration of glycated haemoglobin (HbA1c).

**Methods:**

Our study population consisted of 140 Iranian adults from seven different occupational groups and a group of non-occupationally exposed female participants. The plasma concentration of PCBs were determined at the laboratory of occupational toxicology at RWTH Aachen University, Germany. We considered an HbA1c concentration of 5.7% and more as indicating a disturbed glucose metabolism. Logistic regression was used to assess the association between quartiles of concentrations of PCB congeners and serum HbA1c.

**Results:**

Participants with an increased HbA1c value had higher plasma levels of PCB 138, 153, 180 and the PCB sum, although this association was statistically not significant. There was no significant difference between the levels of PCB 138, 153, 180, the sum of these congeners, and PCB 118 in their quartiles when comparing with HbA1c concentrations.

**Conclusions:**

For our cohort, we could not demonstrate a significant association between PCB and HbA1c concentrations indicating a disturbance of glucose metabolism.

**Electronic supplementary material:**

The online version of this article (10.1186/s12995-018-0199-4) contains supplementary material, which is available to authorized users.

## Background

Diabetes mellitus (DM) is a common non-communicable disease and a leading cause of death worldwide with more than one million casualties in 2010 [1]. According to the International Diabetes Federation (IDF), in 2011 366 million cases of diabetes (8.3% adults) were reported worldwide. In Iran the prevalence of the disease was 11.4% of the total population in 2011, representing an increase of about 35% since 2005 [2]. A further mean increase of 194,000 DM patients per year among the Iranian adult population aged 20–79 years is projected [3]. In addition to patients suffering from clinically manifest DM there is an unknown number of individuals with glycated haemoglobin (HbA1c) values between 5.7 and 6.4% indicating the status of pre-diabetes which is known to significantly increase the risk of developing type 2 diabetes mellitus (T2DM) and cardiovascular complications [4]. The rapidly increasing incidence of T2DM around the world calls for a better understanding of the role of environmental and occupational pollutants such as the ubiquitous polychlorinated biphenyls (PCB) as potential additional risk factors, along with known factors such as obesity, sedentary life style, genetic predisposition and smoking [5–8].

PCBs are a form of halogenated organic compounds that had been widely used as additives in hydraulic fluids, industrial capacitors, transformers, and as plasticizers for expansion joints until approximately three decades ago [9]. Due to the long half-lives of certain PCB congeners, exposure to these compounds continues through food chains, indoor air pollution and at certain industrial plants and workplaces. In addition, occupational contact with PCB is still common during maintenance and de-installation of electrical equipment manufactured in the past [10–12].

Recently, numerous studies assessed the association between PCB exposure and the development of diabetes and pre-diabetes, which was confirmed with some results particularly for dioxin-like PCB congeners [12–16]. There were also reports of a positive association between increased blood glucose levels and the contamination with non-dioxin-like higher-chlorinated PCB congeners [17–20]. Other studies found no such association between serum PCB and diabetes development [21]. However, results of experimental studies suggested possible mechanisms linking glucose metabolism disorders and PCB exposure. They found that certain PCB congeners may act as endocrine disrupters [22], exacerbate insulin resistance [23] and cause depletion of beta cells [24]. Our study presented here aimed to analyse the association between the serum concentration of PCB and increased levels of blood glucose indicated by the HbA1c level.

## Methods

**Fig. 1:**
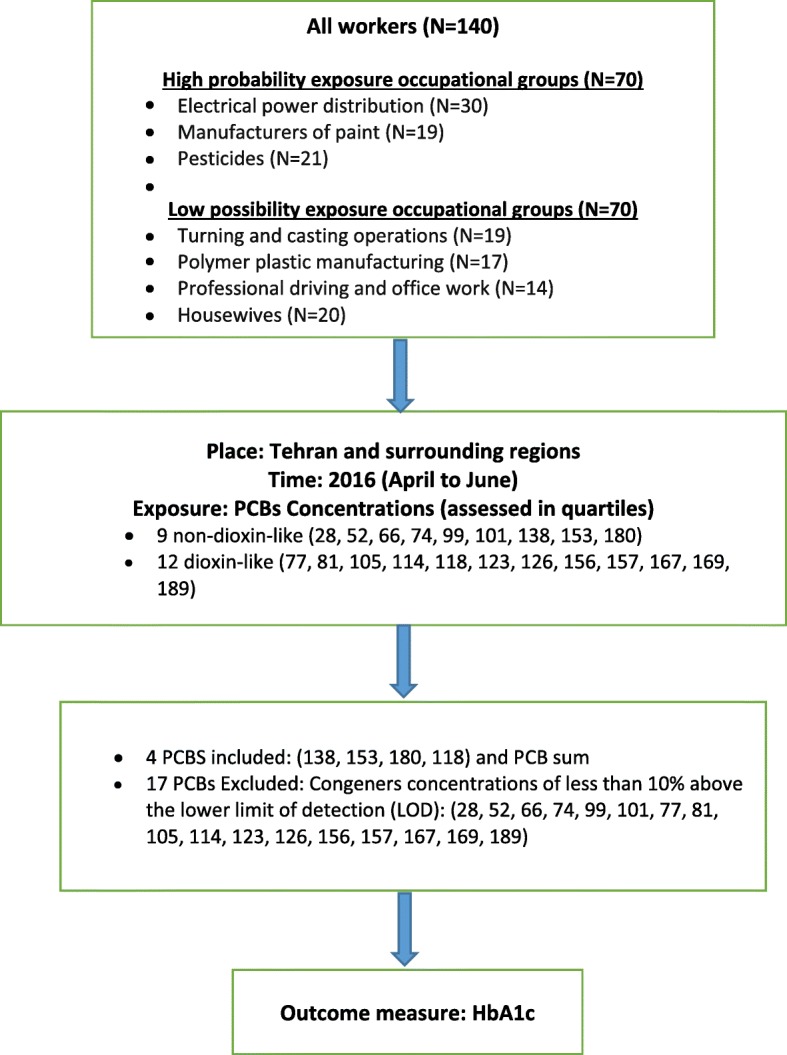
Project outline

The participants of the study were recruited among the employees of six companies in the Tehran region, each representing an industry with an assumedly high or low exposure level of PCB. The employees of the companies and the group of non-occupationally exposed housewives had been selected according to their willingness to participate and good accessibility for setting up the collection of samples. High exposure industries were represented by an electrical power distribution company and manufacturers of paint and pesticides, whereas turning and casting operations, polymer plastic manufacturing and professional driving and office work were considered as low exposure occupations for PCB. „After introducing the study to the company management and their agreement to cooperate, all employees were informed verbally and by notice board on the project, and invited to participate voluntarily. As the allocation to the different professional groups was done only afterwards on the basis of the interviews, a systematic bias by filtering the participants was largely avoided. After excluding missing data for HbA1c or cases with missing biomonitoring (6 samples were damaged during transport to Germany), 140 cases remained. The distribution among the groups is shown in Fig. 1.

A single interviewer collected all data on demographic status, diet habits and occupational history, and measured the participants’ height and weight using calibrated standard devices. All serum samples, obtained from approximately five ml venous blood, were analysed in the same laboratory in Tehran for HbA1c concentrations and lipid profiles. Plasma samples were shipped by commercial mailing service to the occupational toxicology laboratory of the Institute of Occupational, Social and Environmental Medicine at RWTH Aachen University, Germany for determining nine non-dioxin-like (28, 52, 66, 74, 99, 101, 138, 153, 180) and 12 dioxin-like (77, 81, 105, 114, 118, 123, 126, 156, 157, 167, 169, 189) PCB congeners by using gas chromatography coupled with mass spectrometry (GC/MS). For a detailed description of the method we refer to Schettgen et al. [25, 26].

Congeners concentrations of less than 10% above the lower limit of detection (LOD) were excluded from statistical analysis. Therefore, the further analyses have been carried out for PCB 138, 153, 180 and 118. In this study we used a LOD of 0.01 μg/l. All results below this threshold were set to 0.005 μg/l. The plasma concentration of PCBs were categorized using their quartile values. The first quartile (25%) was used as the reference category. If more than 25% of the values of congeners were below LOD, all these values were added to the first quartile. The remaining values were divided into three equal groups forming the quartiles two to four [9]. Following current recommendations by the American Diabetes Association, we used the serum concentration of HbA1c (5.7% or more) for the diagnosis of diabetes or pre-diabetes. Using the HbA1c concentration had several advantages compared with other laboratory parameters, such as fasting plasma glucose or the oral glucose tolerance test, including greater convenience as pre-test fasting was not required, a better pre-analytical stability, and less day-to-day perturbations during stress and illness [27].

We categorized the participants metabolic disorders status according to the HbA1c level, as it is more reliable while some of them might not adhere to the restrict recommended fasting period despite prior rigorous advice.

### Statistical tests

For statistical analysis we used the IBM SPSS Statistics 22 package. Comparing variable distributions with normal distribution curve was done by using the *Kolmogorov–Smirnov test*. A *P*-value of less than 0.05 was considered significant for all statistical tests. Logistic regression modelling was used to evaluate the association between diabetes, pre-diabetes and serum concentrations of PCB congeners, as well as the other considered covariates (age, gender, BMI and total lipid concentration). Adjusted odds ratios (ORs) for these covariates and their corresponding 95% confidence intervals (CIs) are reported. Total serum lipid was calculated using the short formula proposed by Phillips: Total lipids (mg/dL) = 2.27*Total cholesterol + TG (triglycerides) + 0.623 [28]. A generalized maximum likelihood Wald X^2^ test was used on dummy variables of four quartiles to evaluate the differences of the average proportion of diabetic cases across the quartiles [29].

### Ethical considerations

All participants of the study gave written informed consent beforehand. Participation in the study was voluntary. Confidentiality was guaranteed through anonymous data gathering. The study was approved by the Ethics Committee at the Research Division of the Ministry of Health in Tehran (Project number: 93–03–103-26,804-144,653).

## Results

Table 1 shows the demographic characteristics and metabolic key values of the 140 examined participants, of which 83% were males. The age range lay between 20 and 71 years (mean 38.6, SD ± 10.38 years). Mean and median BMI were 26.41 and 26.34 kg/m^2^ respectively and thus falling into the “overweight” category (BMI between 25 and 30 kg/m^2^) [30]. Of the 140 participants, 77 (55%) were classified as being diabetic or pre-diabetic, and were also older than the participants with normal HbA1c values (*P* = 0.002). In addition, they had higher values of PCB 138, 153, 180 and the PCB sum, although this was not statistically significant.

**Table 1:** Demographic and metabolic characteristics of the two blood glucose status groups, as determined by HbA1c level

		Total (*N* = 140)	Metabolic disorder (HbA1C ≥ 5.7) (*N* = 77)	Without Metabolic disorder (HbA1C < 5.7) (*N* = 63)	*P* value
Gender	N (%)				
	Male	116 (82.9)	62 (53.4)	54 (46.6)	0.42
	Female	24 (17.1)	15 (62.5)	9 (37.5)	
Age	[Year] Mean (Sd)	38.61 (10.38)	41.04 (11.04)	35.63 (8.7)	0.002^*^
BMI Mean	[Kg/M^2^] (Sd)	26.41 (3.76)	26.52 (3.62)	26.26 (3.96)	0.69
HbA1c Mean	[%] (Sd)	5.86 (1.12)	6.28 (1.35)	5.34 (0.27)	< 0.001^*^
FBS Mean	[Mg/Dl] (Sd)	99.90 (37.02)	106.31 (47.89)	92.27 (13.52)	0.03^*^
Total Lipid Mean	[Mg/Dl] (Sd)	577.09 (162.91)	583.70 (151.26)	569.09 (176.90)	0.30

*BMI* Body Mass Index, *HbA1c* glycated hemoglobin, *FBG* Fasting Blood Glucose

*Statistically significant relation (*P* value < 0.05)

The mean plasma concentrations of PCB 138, 153, 180, 118 and the PCB sum, as well as their 25th, 50th and 75th percentiles, subdivided in two groups (HbA1c ≥ 5.7% and < 5.7%) are shown in Table 2.

**Table 2:** Distribution of PCB 138, 153, 180, sum, and 118 in two blood glucose status groups, determined by HbA1c level

	Total (N = 140)					Metabolic disorder (HbA1C ≥ 5.7) (N = 77)					Without Metabolic disorder (HbA1C < 5.7) (N = 63)				
	PCB 138 [μg/l]	PCB 153 [μg/l]	PCB 180 [μg/l]	PCB sum [μg/l]	PCB 118 [μg/l]	PCB 138 [μg/l]	PCB 153 [μg/l]	PCB 180 [μg/l]	PCB sum [μg/l]	PCB 118 [μg/l]	PCB 138 [μg/l]	PCB 153 [μg/l]	PCB 180 [μg/l]	PCB sum [μg/l]	PCB 118 [μg/l]
Mean	0.026	0.038	0.027	0.107	0.009	0.029	0.043	0.032	0.120	0.009	0.023	0.032	0.020	0.090	0.010
SD	0.03	0.03	0.03	0.08	0.01	0.03	0.04	0.03	0.10	0.01	0.02	0.03	0.02	0.07	0.01
Minimum	0.005	0.005	0.005	0.030	0.005	0.005	0.005	0.005	0.030	0.005	0.005	0.005	0.005	0.030	0.005
Percentile 25	0.005	0.016	0.005	0.050	0.005	0.008	0.020	0.005	0.058	0.005	0.005	0.014	0.005	0.039	0.005
Median	0.020	0.032	0.017	0.082	0.005	0.023	0.034	0.021	0.09	0.005	0.014	0.027	0.014	0.070	0.005
Percentile 75	0.036	0.053	0.039	0.140	0.009	0.037	0.053	0.047	0.156	0.011	0.033	0.051	0.033	0.132	0.006
Maximum	0.171	0.170	0.173	0.450	0.109	0.171	0.170	0.173	0.450	0.068	0.154	0.151	0.069	0.393	0.109

There was no statistically significant difference of the PCB congener levels between the two groups

The relationship between the concentrations of PCB 138, 153, 180, the PCB sum, and PCB 118 in different quartiles and according to blood glucose status is shown in Table 3. The first quartile was used as the reference group. The third quartiles of the values for PCB 138 and 153 were significantly increased (*P* = 0.01) compared to the reference group. Regarding the relationship in other quartiles, only PCB 153 in the second quartile and PCB 180 in the fourth quartile showed statistically significant differences in both glucose status groups (*P* = 0.03 and 0.01 respectively). The highest ORs were found for the third quartiles of PCB153 and PCB138 (OR = 3.51 and 3.44, respectively). As there was no difference of the categorized HbA1c levels among the PCB 118 quartiles, and a large number of participants fell into the first quartile, the same analysis with dichotomous PCB 118 values (LOD 0.005 as cut off) was performed. This analysis showed that 20 (55.6%) participants with a PCB 118 level above LOD had also an increased HbA1c level of 5.7% or more, compared to 57 (54.8%) in the other group with no detected PCB 118 (*P* = 0.94, OR = 1.03 CI 95%: 0.48–2.21).

**Table 3:** The relationship between level of PCB 138, 153, 180, sum, and 118 in different quartiles and blood glucose status (divided into 2 groups considering HBA1c = 5.7% as cut-off), considering the first quartile as the reference group (unadjusted ORs)

Variables	Quartile 1	Quartile 2	Quartile 3	Quartile 4
PCB138 N (HbA1c ≥ 5.7%)	47 (19)	31 (18)	30 (21)	32 (19)
OR (CI)	1.0 (Ref)	2.04 (0.81–5.12)	3.44 (1.30–9.11)	2.15 (0.86–5.38)
*P* value	–	0.13	0.01^*^	0.10
PCB153 N (HbA1c ≥ 5.7%)	34 (12)	36 (22)	35 (23)	35 (20)
OR (CI)	1.0 (Ref)	2.88 (1.09–7.61)	3.51 (1.30–9.46)	2.44 (0.93–6.45)
*P* value	–	0.03^*^	0.01^*^	0.07
PCB180 N (HbA1c ≥ 5.7%)	51 (23)	30 (18)	29 (14)	30 (22)
OR (CI)	1.0 (Ref)	1.83 (0.73–4.56)	1.14 (0.46–2.83)	3.35 (1.26–8.91)
*P* value	–	0.19	0.78	0.01^*^
PCB sum^a^ N (HbA1c ≥ 5.7%)	36 (15)	34 (20)	35 (20)	35 (22)
OR (CI)	1.0 (Ref)	2 (0.77–5.18)	1.87 (0.73–4.79)	2.37 (0.91–6.15)
P value	–	0.15	0.19	0.07
PCB118 N (HbA1c ≥ 5.7%)	104 (57)	12 (6)	12 (7)	12 (7)
OR (CI)	1.0 (Ref)	0.82 (0.25–2.73)	1.15 (0.34–3.87)	1.15 (0.34–3.87)
*P* value	–	0.75	0.82	0.82

^a^Calculated as sum of PCB 28 + 52 + 101 + 138 + 153 + 180 according to the German PCB-guideline [37, 38]

*Statistically significant relation (*P* value < 0.05)

The relationship between PCB levels and indications of a disturbed of blood glucose is shown in Table 4. In the logistic regression, adjusted for age, gender, BMI and total lipid concentrations, none of the pairwise differences between the first quartile and the remaining 2nd, 3rd and 4th quartiles reached statistical significance.

**Table 4:** Logistic regression, model adjusted for sex, age, BMI, total lipid assessing the relationship between level of PCB 138, 153, 180, sum, and 118 in different quartiles and blood glucose status (divided into 2 groups considering HBA1c = 5.7% as cut-off), considering the first quartile as the reference group

Variables		Quartile 1	Quartile 2	Quartile 3	Quartile 4	Wald χ2 *p*-value
PCB138	Exp (B)	1.0	1.52	2.44	1.12	0.34
	*P* Value	–	0.44	0.09	0.85	
PCB153	Exp (B)	1.0	2.45	2.55	1.00	0.12
	*P* Value	–	0.09	0.08	1	
PCB180	Exp (B)	1.0	1.23	0.72	1.66	0.53
	*P* Value	–	0.69	0.53	0.39	
PCB sum^a^	Exp (B)	1.0	1.38	1.25	0.93	0.87
	*P* Value	–	0.54	0.68	0.91	
PCB118	Exp (B)	1.0	0.62	0.87	0.47	0.73
	*P* Value	–	0.48	0.82	0.32	

^a^Calculated as sum of PCB 28 + 52 + 101 + 138 + 153 + 180 according to the German PCB-guideline [37, 38]

## Discussion

In this study we investigated 140 participants categorized in two groups according to their HbA1c level, either indicating a disturbed glucose metabolism or showing normal values of less than 5.7%. For our cohort we could not find a significant association between plasma PCB levels and HbA1c values. As expected, age as one of the other tested co-variables, had the strongest effect on HbA1c. We have also found that the PCB-burden of our study population was lower in comparison with similar study groups [31].

The high number of male participants in this study was due to the fact that the study population originated from an occupational health investigation among workers from industrial workplaces. In Iran the majority of the working population particularly in the manufacturing and power industry is male. In view of the overall low PCB burden, however, an influence of gender on the results can be denied, especially since the regression has been adjusted for gender. Published results of a study among the American native population suggested that the strongest association with diabetes was due to the lower-chlorinated PCB congeners, which are less persistent in the human body [32]. In our study group, for none of the low chlorinated PCB congeners more than 10% of the measured plasma values lay above the LOD. Therefore we had to exclude the results for these congeners, which had probably the strongest effect on glucose metabolism, from further analysis. This together with the small sample size and the limited overall PCB burden may explain the discrepancy between our results and other cross sectional studies showing a strong dose-response relationship between serum concentrations of PCB 153 and diabetes [17, 31]. We can still say that these findings are not contradicting our results, although the dose response relationship lost statistical significance after adjustment for the co-variables age, gender, BMI and serum total lipid in the regression model. Considering the strong influence of age on the plasma levels of higher chlorinated PCBs [25], the reduced observed relationship in the statistical model may be contributed primarily to the effect of age.

In 2010, Lee et al. showed a non-linear U-shaped relation between high chlorinated PCBs and diabetes risk [16]. Their separate analysis of the different PCB congeners indicated that the blood concentrations of PCB 74, 118 and 126 (dioxin like PCBs) as well as PCB 138, 153, 170, 180 and 187 (non-dioxin like PCBs) were associated with signs of the metabolic syndrome. This association was much stronger between lower concentrations than higher background concentrations [16], which was explained by the role of PCB as an endocrine disrupter. High-dose exposure may lead to down-regulation of receptors as the dose further increases [33]. This finding may warrant the conclusion that exposure to low doses of PCB can be at least as harmful as to higher doses [34, 35]. However, in the present study, we could not investigate low chlorinated congeners, such as PCB 74 and 126, since less than 10% of the studied population had serum concentrations above the LOD. Although the analysis of PCB 138 and 153 showed some effect on the glucose metabolism in the second and third quartiles, this association did not reach a statistically significant level.

The results of our study did not demonstrate any association between the serum values of PCB 118 and the PCB sum on the one hand and the risk of developing a disturbed glucose metabolism on the other, which is inconsistent with previous studies [16, 36]. A large Japanese study demonstrated a significantly higher prevalence of DM at the third and fourth quartiles of dioxin like PCBs [13]. That may be due to the fact that PCBs plasma levels of the Iranian population are generally low. We must however also keep in mind that we could not analyse the association of all dioxin like PCBs as mentioned formerly.

This study‘s major strength was the use of HbA1c rather than e.g. fasting blood glucose as an indicator of an unbalanced glucose metabolism or manifest diabetes. The assessment of a specific additional effect of raised PCB levels on the glucose metabolism was further validated in our participants by controlling for other important risk factors. Another important strength was the inclusion of different occupational groups with a wide range of assumed PCB-exposure risks, together with the housewives as a non-occupationally exposed reference group and possibly indicating the background exposure of a typical urban population. This was an advantage over previous studies on PCB only assessing subjects with known high exposures to various pollutants in occupational settings, and thus avoiding a selection bias.

Due to the cross sectional design of our study and the anonymity of all participants, only one contact could be made to collect blood samples and record demographic data. Therefore it was not possible to assess incidence and changes over time in exposed and non-exposed subgroups. The low overall levels of dioxin-like PCBs, except for PCB 118, made it impossible to assess their association with increased glucose levels and diabetes. Our attempt to consider the effect of each PCB congener separately, rather than building groups of similar types, hampered significant results.

## Conclusion

For our study cohort of male industrial workers and not-occupationally exposed housewives we could not demonstrate a significant effect of different PCB congeners on the concentration of HbA1c.

## Additional file

Additional file 1: PCB_DM_data. (XLSX 55 kb)

## Abbreviations

ADA: American Diabetes Association
CI: Confidence interval
DM: Diabetes mellitus
FPG: Fasting plasma glucose
HbA1c: % of glycosylated haemoglobin
IDF: International Diabetes Federation
LOD: Limit of detection
OGTT: Oral glucose tolerance test
ORs: Adjusted odds ratios
PCB: Polychlorinated biphenyls
POPs: Persistent organic pollutants
RWTH: Rheinisch-Westfaelische Technische Hochschule / rhenish-westphalian technical University
T2DM: Type 2 diabetes mellitus
